# Content Validity Assessment of a Newly Developed Emergency Medical Dispatch and Triage Protocol in Thailand

**DOI:** 10.3390/jcm14197125

**Published:** 2025-10-09

**Authors:** Thongpitak Huabbangyang, Duangpon Thepmanee, Phudit Buaprasert, Pit Chansomboon, Jiraporn Sri-on, Rapeeporn Rojsaengroeng

**Affiliations:** 1 Department of Disaster and Emergency Medical Operation, Faculty of Science and Health Technology, Navamindradhiraj University, Bangkok 10300, Thailand; thongpitak@nmu.ac.th (T.H.); duangporn@nmu.ac.th (D.T.); 2 Department of Emergency Medicine, Emory University School of Medicine, Atlanta, GA 30322, USA; phudit.buaprasert@gmail.com; 3 Department of Emergency Medicine, Udonthani Hospital, Udonthani 41000, Thailand; pit8735@gmail.com; 4 Department of Emergency Medicine, Faculty of Medicine, Vajira Hospital, Navamindradhiraj University, Bangkok 10300, Thailand; jiraporn.rew@gmail.com

**Keywords:** emergency medical dispatch, emergency medical services, EMS communication systems, medical triage protocols, prehospital emergency care

## Abstract

**Background/Objectives:** Accurate telephone triage of emergency medical cases plays a crucial role in improving outcomes for critically ill patients. Effective triage enables emergency medical dispatchers to provide appropriate pre-arrival instructions and to deploy operational units according to the patient’s severity level. This study aimed to develop and assess the content validity of the Emergency Medical Triage Protocol and Criteria-Based Dispatch Code (EMTP-CBDC) for Thailand. The objective was to ensure the tool’s content accuracy and applicability in prioritizing emergency responses in line with medical urgency, considering global changes and universal standards. **Methods**: A cross-sectional descriptive study was conducted from 15–30 April 2024. The content validity of the newly developed EMTP-CBDC, comprising 30 symptom groups, was evaluated by five emergency physician experts with at least 1 year of experience in emergency medical oversight. The assessment focused on four aspects: relevance, clarity, simplicity, and ambiguity. The Content Validity Index (CVI) was calculated at both the item level (I-CVI) and the scale level using the average index (S-CVI/Ave). To adjust for chance agreement, the probability of chance agreement (Pc) and the modified kappa coefficient (k*) were calculated for each item. **Results**: The content validation revealed I-CVI values ranging from 0.80 to 1.00 across all items. The S-CVI/Ave scores were 0.97 for relevance, 0.93 for clarity, 0.98 for simplicity, and 0.94 for ambiguity. These values surpassed the accepted thresholds for content validity. **Conclusions**: The EMTP-CBDC developed for Thailand demonstrated good content validity across relevance, clarity, simplicity, and ambiguity. Further studies are needed to establish its reliability and field performance before routine implementation.

## 1. Introduction

The enhancement of efficient and effective emergency medical services (EMSs) requires a systematic approach aligned with the core principles of emergency medicine, as represented by the Star of Life [1,2]. One of the essential components of EMS is the emergency medical dispatcher (EMD), who conducts telephone triage using standardized emergency medical triage protocols. Several high-income countries have developed and implemented comprehensive triage systems to support accurate dispatch and pre-arrival instructions (PAIs). For example, the Netherlands uses the Dutch Field Triage Protocol (National Protocol of Ambulance Services), developed in accordance with the American College of Surgeons Committee on Trauma (ACS-COT) guidelines [3]. Similarly, the United States [4,5], the United Kingdom [6,7], and Australia [8,9] employ the Medical Priority Dispatch System (MPDS), which includes triage codes for 33 symptom groups.

A systematic review evaluating the effectiveness of MPDS in EMS systems found that its implementation was associated with improved patient outcomes, particularly through reduced response times [10]. In Norway, the Norwegian Index for Medical Dispatch is used to evaluate and triage patients [11,12], while Japan utilizes protocols issued by the Fire and Disaster Management Agency [13,14]. In Thailand, the original criteria-based dispatch (CBD) codes covered 26 symptom groups [15]. These systems provide EMDs with the ability to offer PAIs—basic, evidence-based medical instructions to callers before emergency responders arrive on the scene [4,5,6,7,8,9,11,12,13,14,15]. A previous population-based retrospective cohort study demonstrated that a combination of EMD-reported information and MPDS codes effectively predicted the severity of traffic injuries, resulting in significantly more accurate ambulance dispatch [16].

In Thailand, the Emergency Medical Act B.E. 2551 governs the structure and operation of emergency services, including oversight by the Emergency Medical Committee and the National Institute for Emergency Medicine (NIEMS) [17]. Under Section 28(1) of this Act, hospitals and EMS units are required to triage and respond to emergency cases based on medical urgency using established emergency medical triage protocols and CBD codes. These tools aim to ensure that patients receive timely and appropriate care based on urgency, in line with national policy and international standards.

Since 2009, Thailand has piloted a CBD system comprising 25 symptom groups, which was later integrated into the Information Technology for Emergency Medical System Version 4.0 [18]. In 2025, the EMS database was upgraded to the Intelligence Digital Emergency Medical Services Platform, allowing EMDs to deliver symptom-specific pre-arrival assistant instruction PAIs [19]. Since 2011, NIEMS has issued CBD codes for each symptom group, categorizing patients by urgency levels—red, yellow, green, and white—to facilitate appropriate resource deployment. However, updates to the CBD system have not kept pace with advances in medical knowledge, evolving emergency conditions, or technological innovations such as automated PAI systems.

Thailand’s emergency operational units are categorized into three tiers—basic, advanced, and specialized [20]. During the COVID-19 pandemic, beginning in 2020, the government designated COVID-19 as an endemic disease [21]. NIEMS subsequently updated the CBD system, adding COVID-19 as the 26th symptom group in March 2022. Despite this addition, challenges in the application of the CBD system persisted, and user feedback indicated that the protocol did not fully meet current needs. To address these gaps, NIEMS appointed a panel of experts to revise the emergency medical triage protocol and CBD system, building upon the foundation established in 2013 and updated in 2021. These efforts aim to align the system with contemporary clinical and technological standards while ensuring usability for frontline personnel.

The present study was conducted to improve and validate the content accuracy of Thailand’s newly revised Emergency Medical Triage Protocol and Criteria-Based Dispatch Code (EMTP-CBDC). The objective was to ensure that the revised system provides accurate, up-to-date guidance for EMDs, supports effective triage based on urgency, and aligns with international best practices.

## 2. Materials and Methods

This cross-sectional descriptive study was conducted from 15 to 30 April 2024. Data collection and reporting followed the Standards for the Reporting of Observational Studies in Epidemiology (STROBE) guidelines [22]. Data were collected from a panel of five emergency physicians selected using a purposive sampling method based on their qualifications and expertise in emergency medicine.

Participants were required to hold a diploma in emergency medicine, have experience in prehospital emergency care, be at least 18 year of age, and have a minimum of 1 year of experience in emergency medical oversight. Physicians who were unwilling or declined to complete the evaluation form were excluded from the study.

The newly developed and improved EMTP-CBDC of Thailand, comprising 30 symptom groups created by the NIEMS, along with a Content Validity Index (CVI) evaluation form, was sent to the experts via email. The evaluation form was administered through Google Forms and assessed four aspects: relevance, clarity, simplicity, and ambiguity, each rated on a 4-point scale: relevance: 1 = *not relevant*, 2 = *needs revision*, 3 = *relevant with minor revision*, 4 = *very relevant*; clarity: 1 = *not clear*, 2 = *needs revision*, 3 = *clear with minor revision*, 4 = *very clear*; simplicity: 1 = *not simple*, 2 = *needs revision*, 3 = *simple with minor revision*, 4 = *very simple*; and ambiguity: 1 = *doubtful*, 2 = *needs revision*, 3 = *no doubt but minor revision needed*, 4 = *meaning is clear* [23].

After evaluation, the item-level CVI (I-CVI) was calculated for each aspect. Items with a mean I-CVI of ≥ 0.80 were considered valid. Items scoring below this threshold were revised by the authors and re-evaluated by the same panel until they met the validity criterion [24]. In addition to numeric ratings, all experts were invited to provide qualitative comments for each item. Items that received an I-CVI < 1.00 or were flagged by experts as unclear, complex, or ambiguous were revised by the research team according to the feedback received. The revised items were then resubmitted to the same panel of experts for re-evaluation until the predetermined threshold of I-CVI ≥ 0.80 was achieved. This iterative process ensured that high CVI values reflected consensus after revision rather than initial agreement alone.

The equations used to calculate the item-level content validity index (I-CVI), the scale-level index (S-CVI/Ave), the probability of chance agreement (Pc), and the modified kappa coefficient (k*), as well as the bootstrap procedure for estimating the 95% confidence interval of S-CVI/Ave, are presented in Appendix A. Data were recorded in Microsoft Excel for further analysis.

Variable definition: The EMTP-CBDC refers to a tool developed by NIEMS for classifying emergency symptoms and dispatching appropriate EMSs. It includes a structured introduction, chief complaint and injury categories, and telephone guidance for critical, life-threatening conditions.

As the primary objective was to validate the EMTP-CBDC using CVI analysis, a sample size of five experts was deemed appropriate based on previous literature recommending a minimum of five experts for such validation studies [23]. Thus, five participants were purposively selected. Content accuracy was assessed using CVI analysis, including both item-level CVI (I-CVI) and the scale-level average index (S-CVI/Ave). To adjust for chance agreement, the probability of chance agreement (Pc) and the modified kappa coefficient (k*) were calculated for each item. The interpretation of k* followed established thresholds (≥0.74 = excellent, 0.60–0.73 = good, 0.40–0.59 = fair, <0.40 = poor) [25]. In addition, 95% confidence intervals (CIs) for S-CVI/Ave were estimated using nonparametric bootstrap resampling with 10,000 iterations. Statistical analysis was performed using IBM SPSS Statistics for Windows, Version 28.0 (IBM Corp., Armonk, NY), and custom scripts for bootstrap calculations.

## 3. Results

### 3.1. Relevance

The evaluation of content accuracy in terms of relevance for the EMTP-CBDC in Thailand showed that the I-CVI values ranged from 0.80 to 1.00 across all 30 items. Items with I-CVI = 1.00 corresponded to unanimous expert agreement (A = 5), yielding Pc = 0.0313 and k* = 1.00 (excellent). Items with I-CVI = 0.80 (A = 4) had Pc = 0.1563 and k* = 0.763, also interpreted as excellent. Overall, the S-CVI/Ave was 0.97 (95% CI: 0.95–0.99), indicating excellent content validity for relevance (Table 1; Table A1).

### 3.2. Clarity

The evaluation of content accuracy in terms of clarity for the EMTP-CBDC in Thailand showed that the I-CVI values also ranged from 0.80 to 1.00. For unanimous items, k* = 1.00 (excellent), and for items with four of five experts agreeing, k* = 0.763 (excellent). The S-CVI/Ave was 0.93 (95% CI: 0.90–0.97). While slightly lower than the other domains, this result still demonstrated good to excellent clarity (Table 2; Table A2).

### 3.3. Simplicity

The evaluation of content accuracy in terms of simplicity for the EMTP-CBDC in Thailand showed that the I-CVI values were between 0.80 and 1.00, with corresponding Pc and k* values consistent with the patterns above (Pc = 0.0313, k* = 1.00 for unanimous agreement; Pc = 0.1563, k* = 0.763 for four-out-of-five agreement). The S-CVI/Ave was 0.98 (95% CI: 0.95–1.00), confirming excellent overall simplicity of the items (Table 3; Table A3).

### 3.4. Ambiguity

The evaluation of content accuracy in terms of ambiguity for the EMTP-CBDC in Thailand showed that the I-CVI values again ranged from 0.80 to 1.00, with Pc and k* identical to the values reported above depending on whether four or five experts agreed. The S-CVI/Ave was 0.94 (95% CI: 0.91–0.97), indicating that the items were sufficiently unambiguous and meeting the predetermined acceptance criteria (Table 4; Table A4).

## 4. Discussion

This study found that the newly improved EMTP-CBDC, Thailand, developed by the NIEM, demonstrated acceptable content validity across all evaluated aspects—relevance, clarity, simplicity, and unambiguity—according to expert assessments. The evaluation encompassed 30 symptom groups and aligned with the development guidelines of the emergency medical system based on the Star of Life principles, which emphasize systematic dispatch and triage beginning with the emergency call via 1669 [1,2]. The findings support the applicability of this updated tool in the Thai context, particularly during a time when the EMSs face evolving challenges. For example, in response to emerging diseases such as COVID-19, the EMS in Thailand added a 26th symptom group, CBC, to its existing classification system in 2022 [22]. This underscores the need to modernize dispatch tools to accommodate dynamic emergency scenarios. When compared with international triage systems—such as the MPDS used in the United States [4,5], England [6,7], and Australia [8,9]—the EMTP-CBDC shows promise in its alignment with global best practices, including the use of symptom group codes and basic telephone instructions by trained personnel (e.g., emergency physicians, paramedics, emergency nurse practitioners, or EMDs).

A previous study involving 43 EMS systems in China reported that incorporating MPDS led to improved diagnostic concordance for acute coronary syndrome between dispatch and on-scene treatment and reduced the time from emergency call to EMS arrival from 20.0 to 16.0 min (*p* < 0.001) [24,26]. Similarly, research on Osaka’s EMS system demonstrated that accurate telephone triage using clearly defined symptom groups reduced unnecessary ambulance usage (from 7.4% to 3.7%) and facilitated more efficient EMS resource allocation through concise PAIs [27].

These findings are consistent with international evidence indicating that accurate, content-validated tools, such as MPDS and the Norwegian Index, help reduce EMS response times and improve dispatch accuracy according to patient severity levels [10,11,12,16]. Although Thailand’s current CBD tool has been in use for over 14 years, it has limitations in terms of modernity and adaptability. This study represents a crucial step toward developing more efficient, standardized tools for national EMS operations and offers a foundation for future improvements.

This study had several important limitations. First, the sample size consisted of only five experts, which may not fully represent the diversity of users at various levels of emergency operations. Additionally, all participants were emergency physicians, which could introduce sample bias and limit the perspectives obtained from other EMS professionals, such as emergency medical dispatchers (EMDs), emergency nurse practitioners, paramedics, and call-takers. The decision to restrict the panel to emergency physicians was made by the development committee of the EMTP-CBDC, appointed by the National Institute for Emergency Medicine (NIEMS) of Thailand, which served as the policy-setting authority and the funding agency for this research. The committee recommended that the initial validation stage be conducted with emergency physicians due to their central role in setting national prehospital standards.

Second, this study focused solely on content validity and did not employ a Delphi consensus process, reliability testing, or field validation. The absence of Delphi rounds and field testing limited the opportunity to refine the tool through iterative consensus and to evaluate its performance in real-world dispatch environments.

Third, the entire validation process was conducted over only two weeks, as determined by the development committee under NIEMS. This limited timeframe was due to administrative and logistical constraints, and although experts were able to complete their evaluations electronically and multiple rounds of revision were undertaken, the short duration may have restricted the thoroughness of the process.

Therefore, future research should incorporate a broader range of stakeholders, extend the validation period, and use methods such as Delphi consensus rounds and field testing to strengthen the tool’s psychometric properties, usability, and generalizability.

## 5. Conclusions

The newly developed EMTP-CBDC for Thailand demonstrated good content validity in terms of relevance, clarity, simplicity, and completeness, based on expert evaluation. While these findings support the tool’s potential suitability for implementation in national dispatch centers and alignment with the needs of a modern EMS system, additional validation is required. In particular, reliability measures (inter-rater and test–retest) and field performance indicators, including dispatch accuracy, adherence to PAIs, and patient outcomes, remain to be established in future studies. To ensure optimal performance in practice, the tool should be tested under operational conditions, undergo continuous review, and be accompanied by structured training programs for call takers and EMDs to promote correct and consistent application.

## Figures and Tables

**Table 1:** Relevance.

Item	Rater1	Rater2	Rater3	Rater4	Rater5	I-CVI
1. Abdominal pain	3	4	4	3	3	1.00
2. Anaphylaxis/Allergic reaction	3	4	4	4	3	1.00
3. Animal bite	3	4	4	3	4	1.00
4. Back pain (non-trauma)	3	4	4	4	2	0.80
5. Bleeding disorder (non-trauma)	4	4	4	4	2	0.80
6. Breathing difficulty	4	4	4	4	3	1.00
7. Cardiac arrest/Death	4	4	4	4	4	1.00
8. Chest discomfort	4	4	4	4	3	1.00
9. Choking	4	4	4	4	4	1.00
10. Convulsion	3	4	4	4	4	1.00
11. Chemical hazard	4	4	4	4	3	1.00
12. Diabetes mellitus	4	4	4	4	3	1.00
13. Eye problems	4	4	4	4	3	1.00
14. Fever	4	4	4	4	3	1.00
15. Headache	2	4	4	4	3	0.80
16. Overdose/Poison	4	4	4	4	4	1.00
17. Psychiatry/Behavior change/Suicide	4	4	4	4	4	1.00
18. Pregnancy/Childbirth	3	4	4	4	3	1.00
19. Pediatric emergency	3	4	4	4	4	1.00
20. Sick person/Unspecify	3	4	4	4	2	0.80
21. Stroke	4	4	4	4	4	1.00
22. Unconsciousness/Fainting	3	4	4	4	4	1.00
23. Assault/Sexual assault	3	4	4	4	4	1.00
24. Burn/Blast	4	4	4	4	3	1.00
25. Drowning/Diving/Water-related injury	4	4	4	4	3	1.00
26. Electrocution	4	4	4	4	3	1.00
27. Falls	3	4	4	4	3	1.00
28. Heat/Cold exposure	3	4	4	4	3	1.00
29. Stab/Gunshot/Penetrating trauma	4	4	4	4	4	1.00
30. Traffic incidents/Vehicle in water	3	3	3	4	4	1.00
S-CVI/Ave					0.97	
Bootstrap 95% CI					0.95–0.99	

**Table 2:** Clarity.

Item	Rater1	Rater2	Rater3	Rater4	Rater5	I-CVI
1. Abdominal pain	3	4	4	3	3	1.00
2. Anaphylaxis/Allergic reaction	3	4	4	3	3	1.00
3. Animal bite	2	4	4	4	3	0.80
4. Back pain (non-trauma)	2	4	4	4	3	0.80
5. Bleeding disorder (non-trauma)	4	4	4	3	2	0.80
6. Breathing difficulty	4	4	4	3	3	1.00
7. Cardiac arrest/Death	3	4	4	4	4	1.00
8. Chest discomfort	3	4	4	2	3	0.80
9. Choking	4	3	3	4	3	1.00
10. Convulsion	3	4	4	4	4	1.00
11. Chemical hazard	4	3	3	4	4	1.00
12. Diabetes mellitus	4	4	4	4	4	1.00
13. Eye problems	3	3	3	3	3	1.00
14. Fever	4	4	4	3	3	1.00
15. Headache	2	4	4	4	3	0.80
16. Overdose/Poison	4	4	4	4	3	1.00
17. Psychiatry/Behavior change/Suicide	4	4	4	2	3	0.80
18. Pregnancy/Childbirth	2	4	4	3	3	0.80
19. Pediatric emergency	3	4	4	4	4	1.00
20. Sick person/Unspecify	3	3	3	4	2	0.80
21. Stroke	4	4	4	4	4	1.00
22. Unconsciousness/Fainting	3	4	4	2	3	0.80
23. Assault/Sexual assault	3	4	4	4	4	1.00
24. Burn/Blast	3	4	4	4	3	1.00
25. Drowning/Diving/Water-related injury	4	3	3	3	3	1.00
26. Electrocution	4	4	4	4	3	1.00
27. Falls	3	4	4	4	3	1.00
28. Heat/Cold exposure	3	4	4	4	3	1.00
29. Stab/Gunshot/Penetrating trauma	4	4	4	3	4	1.00
30. Traffic incidents/Vehicle in water	2	4	4	4	3	0.80
S-CVI/Ave					0.93	
Bootstrap 95% CI					0.90–0.97	

**Table 3:** Simplicity.

Item	Rater1	Rater2	Rater3	Rater4	Rater5	I-CVI
1. Abdominal pain	3	3	3	4	3	1.00
2. Anaphylaxis/Allergic reaction	3	3	3	4	3	1.00
3. Animal bite	2	3	3	4	3	0.80
4. Back pain (non-trauma)	4	4	4	4	3	1.00
5. Bleeding disorder (non-trauma)	4	3	3	4	3	1.00
6. Breathing difficulty	4	3	3	4	3	1.00
7. Cardiac arrest/Death	4	4	4	4	3	1.00
8. Chest discomfort	4	4	4	4	3	1.00
9. Choking	4	4	4	4	3	1.00
10. Convulsion	3	4	4	4	3	1.00
11. Chemical hazard	4	4	4	4	3	1.00
12. Diabetes mellitus	4	4	4	4	3	1.00
13. Eye problems	4	3	3	4	3	1.00
14. Fever	4	4	4	4	3	1.00
15. Headache	3	3	3	4	3	1.00
16. Overdose/Poison	4	4	4	4	4	1.00
17. Psychiatry/Behavior change/Suicide	4	3	3	4	3	1.00
18. Pregnancy/Childbirth	3	4	4	4	3	1.00
19. Pediatric emergency	2	4	4	4	4	0.80
20. Sick person/Unspecify	3	3	3	4	3	1.00
21. Stroke	4	4	4	4	4	1.00
22. Unconsciousness/Fainting	2	4	4	4	4	0.80
23. Assault/Sexual assault	3	4	4	4	4	1.00
24. Burn/Blast	4	4	4	4	3	1.00
25. Drowning/Diving/Water-related injury	4	4	4	4	3	1.00
26. Electrocution	4	4	4	4	3	1.00
27. Falls	3	4	4	4	3	1.00
28. Heat/Cold exposure	3	4	4	4	3	1.00
29. Stab/Gunshot/Penetrating trauma	4	4	4	4	4	1.00
30. Traffic incidents/Vehicle in water	3	4	4	4	3	1.00
S-CVI/Ave					0.98	
Bootstrap 95% CI					0.95–1.00	

**Table 4:** Ambiguity.

Item	Rater1	Rater2	Rater3	Rater4	Rater5	I-CVI
1. Abdominal pain	3	4	4	4	3	1.00
2. Anaphylaxis/Allergic reaction	3	4	4	3	3	1.00
3. Animal bite	2	4	4	4	3	0.80
4. Back pain (non-trauma)	3	4	4	4	3	1.00
5. Bleeding disorder (non-trauma)	4	4	4	4	2	0.80
6. Breathing difficulty	4	4	4	4	3	1.00
7. Cardiac arrest/Death	4	4	4	4	3	1.00
8. Chest discomfort	4	4	4	3	2	0.80
9. Choking	4	4	4	3	3	1.00
10. Convulsion	3	4	4	4	3	1.00
11. Chemical hazard	4	4	4	4	2	0.80
12. Diabetes mellitus	4	4	4	3	3	1.00
13. Eye problems	3	3	3	4	3	1.00
14. Fever	4	4	4	4	3	1.00
15. Headache	3	4	4	4	3	1.00
16. Overdose/Poison	4	4	4	4	3	1.00
17. Psychiatry/Behavior change/Suicide	4	3	3	3	3	1.00
18. Pregnancy/Childbirth	2	4	4	3	3	0.80
19. Pediatric emergency	3	4	4	4	3	1.00
20. Sick person/Unspecify	3	4	4	4	3	1.00
21. Stroke	4	4	4	4	3	1.00
22. Unconsciousness/Fainting	2	4	4	4	3	0.80
23. Assault/Sexual assault	3	4	4	4	4	1.00
24. Burn/Blast	4	4	4	4	2	0.80
25. Drowning/Diving/Water-related injury	4	4	4	3	3	1.00
26. Electrocution	4	4	4	4	3	1.00
27. Falls	3	4	4	4	2	0.80
28. Heat/Cold exposure	3	4	4	4	3	1.00
29. Stab/Gunshot/Penetrating trauma	4	4	4	4	4	1.00
30. Traffic incidents/Vehicle in water	2	4	4	4	3	0.80
S-CVI/Ave					0.94	
Bootstrap 95% CI					0.91–0.97	

## Data Availability

The datasets used and analyzed during the current study are available from the corresponding author on reasonable request.

## Abbreviations

I-CVI	Item-Level Content Validity Index
S-CVI	Scale-Level Content Validity Index
CVI	Content Validity Index
EMTP-CBDC	Emergency Medical Triage Protocol and Criteria-Based Dispatch Code
EMSs	emergency medical services
PAIs	pre-arrival instructions
ACS-COT	American College of Surgeons Committee on Trauma
MPDS	Medical Priority Dispatch System
CBD	criteria-based dispatch
EMDs	emergency medical dispatchers
NIEMS	National Institute for Emergency Medicine
Pc	probability of chance agreement
k*	modified kappa
95%CI	95% confidence interval

## Appendix A

Item-level CVI (I-CVI): I-CVI_j = A_j/N

where A_j is the number of experts rating item j as relevant, and N = 5.

Scale-level CVI: S-CVI = Σ 1(I-CVI_j = 1)/k

S-CVI/Ave = (1/k) Σ I-CVI_j

where k is the total number of items.

Probability of chance agreement (Pc): Pc = C(N, A) × (0.5^N)

where C(N, A) is the binomial coefficient.

Modified kappa (k*): k* = (I-CVI − Pc)/(1 − Pc)

95% CI for S-CVI/Ave (Bootstrap method): S-CVI/Ave = (1/k) Σ I-CVI_j

Bootstrap procedure:

(1) Sample k items with replacement from {I-CVI_1,…,I-CVI_k}.
(2) Compute S-CVI/Ave for each resample.
(3) Repeat B = 10,000 times.
(4) Take 2.5th and 97.5th percentiles of bootstrap distribution as the 95% CI.

## Appendix B

**Table A1:** Relevance.

Item	A (out of 5)	I-CVI	Pc	k*	Interpretation
1. Abdominal pain	5	1.00	0.03125	1.000	Excellent
2. Anaphylaxis/Allergic reaction	5	1.00	0.03125	1.000	Excellent
3. Animal bite	5	1.00	0.03125	1.000	Excellent
4. Back pain (non-trauma)	4	0.80	0.15625	0.763	Excellent
5. Bleeding disorder (non-trauma)	4	0.80	0.15625	0.763	Excellent
6. Breathing difficulty	5	1.00	0.03125	1.000	Excellent
7. Cardiac arrest/Death	5	1.00	0.03125	1.000	Excellent
8. Chest discomfort	5	1.00	0.03125	1.000	Excellent
9. Choking	5	1.00	0.03125	1.000	Excellent
10. Convulsion	5	1.00	0.03125	1.000	Excellent
11. Chemical hazard	5	1.00	0.03125	1.000	Excellent
12. Diabetes mellitus	5	1.00	0.03125	1.000	Excellent
13. Eye problems	5	1.00	0.03125	1.000	Excellent
14. Fever	5	1.00	0.03125	1.000	Excellent
15. Headache	4	0.80	0.15625	0.763	Excellent
16. Overdose/Poison	5	1.00	0.03125	1.000	Excellent
17. Psychiatry/Behavior change/Suicide	5	1.00	0.03125	1.000	Excellent
18. Pregnancy/Childbirth	5	1.00	0.03125	1.000	Excellent
19. Pediatric emergency	5	1.00	0.03125	1.000	Excellent
20. Sick person/Unspecify	4	0.80	0.15625	0.763	Excellent
21. Stroke	5	1.00	0.03125	1.000	Excellent
22. Unconsciousness/Fainting	5	1.00	0.03125	1.000	Excellent
23. Assault/Sexual assault	5	1.00	0.03125	1.000	Excellent
24. Burn/Blast	5	1.00	0.03125	1.000	Excellent
25. Drowning/Diving/Water-related injury	5	1.00	0.03125	1.000	Excellent
26. Electrocution	5	1.00	0.03125	1.000	Excellent
27. Falls	5	1.00	0.03125	1.000	Excellent
28. Heat/Cold exposure	5	1.00	0.03125	1.000	Excellent
29. Stab/Gunshot/Penetrating trauma	5	1.00	0.03125	1.000	Excellent
30. Traffic incidents/Vehicle in water	5	1.00	0.03125	1.000	Excellent

Abbreviations: A = number of experts rating relevant; Pc = probability of chance agreement; k* = modified kappa; I-CVI = Item-Level Content Validity Index.

**Table A2:** Clarity.

Item	A (out of 5)	I-CVI	Pc	k*	Interpretation
1. Abdominal pain	5	1.00	0.03125	1.000	Excellent
2. Anaphylaxis/Allergic reaction	5	1.00	0.03125	1.000	Excellent
3. Animal bite	4	0.80	0.15625	0.763	Excellent
4. Back pain (non-trauma)	4	0.80	0.15625	0.763	Excellent
5. Bleeding disorder (non-trauma)	4	0.80	0.15625	0.763	Excellent
6. Breathing difficulty	5	1.00	0.03125	1.000	Excellent
7. Cardiac arrest/Death	5	1.00	0.03125	1.000	Excellent
8. Chest discomfort	4	0.80	0.15625	0.763	Excellent
9. Choking	5	1.00	0.03125	1.000	Excellent
10. Convulsion	5	1.00	0.03125	1.000	Excellent
11. Chemical hazard	5	1.00	0.03125	1.000	Excellent
12. Diabetes mellitus	5	1.00	0.03125	1.000	Excellent
13. Eye problems	5	1.00	0.03125	1.000	Excellent
14. Fever	5	1.00	0.03125	1.000	Excellent
15. Headache	4	0.80	0.15625	0.763	Excellent
16. Overdose/Poison	5	1.00	0.03125	1.000	Excellent
17. Psychiatry/Behavior change/Suicide	4	0.80	0.15625	0.763	Excellent
18. Pregnancy/Childbirth	4	0.80	0.15625	0.763	Excellent
19. Pediatric emergency	5	1.00	0.03125	1.000	Excellent
20. Sick person/Unspecify	4	0.80	0.15625	0.763	Excellent
21. Stroke	5	1.00	0.03125	1.000	Excellent
22. Unconsciousness/Fainting	4	0.80	0.15625	0.763	Excellent
23. Assault/Sexual assault	5	1.00	0.03125	1.000	Excellent
24. Burn/Blast	5	1.00	0.03125	1.000	Excellent
25. Drowning/Diving/Water-related injury	5	1.00	0.03125	1.000	Excellent
26. Electrocution	5	1.00	0.03125	1.000	Excellent
27. Falls	5	1.00	0.03125	1.000	Excellent
28. Heat/Cold exposure	5	1.00	0.03125	1.000	Excellent
29. Stab/Gunshot/Penetrating trauma	5	1.00	0.03125	1.000	Excellent
30. Traffic incidents/Vehicle in water	4	0.80	0.15625	0.763	Excellent

Abbreviations: A = number of experts rating relevant; Pc = probability of chance agreement; k* = modified kappa; I-CVI = Item-Level Content Validity Index.

**Table A3:** Simplicity.

Item	A (out of 5)	I-CVI	Pc	k*	Interpretation
1. Abdominal pain	5	1.00	0.03125	1.000	Excellent
2. Anaphylaxis/Allergic reaction	5	1.00	0.03125	1.000	Excellent
3. Animal bite	4	0.80	0.15625	0.763	Excellent
4. Back pain (non-trauma)	5	1.00	0.03125	1.000	Excellent
5. Bleeding disorder (non-trauma)	5	1.00	0.03125	1.000	Excellent
6. Breathing difficulty	5	1.00	0.03125	1.000	Excellent
7. Cardiac arrest/Death	5	1.00	0.03125	1.000	Excellent
8. Chest discomfort	5	1.00	0.03125	1.000	Excellent
9. Choking	5	1.00	0.03125	1.000	Excellent
10. Convulsion	5	1.00	0.03125	1.000	Excellent
11. Chemical hazard	5	1.00	0.03125	1.000	Excellent
12. Diabetes mellitus	5	1.00	0.03125	1.000	Excellent
13. Eye problems	5	1.00	0.03125	1.000	Excellent
14. Fever	5	1.00	0.03125	1.000	Excellent
15. Headache	5	1.00	0.03125	1.000	Excellent
16. Overdose/Poison	5	1.00	0.03125	1.000	Excellent
17. Psychiatry/Behavior change/Suicide	5	1.00	0.03125	1.000	Excellent
18. Pregnancy/Childbirth	5	1.00	0.03125	1.000	Excellent
19. Pediatric emergency	4	0.80	0.15625	0.763	Excellent
20. Sick person/Unspecify	5	1.00	0.03125	1.000	Excellent
21. Stroke	5	1.00	0.03125	1.000	Excellent
22. Unconsciousness/Fainting	4	0.80	0.15625	0.763	Excellent
23. Assault/Sexual assault	5	1.00	0.03125	1.000	Excellent
24. Burn/Blast	5	1.00	0.03125	1.000	Excellent
25. Drowning/Diving/Water-related injury	5	1.00	0.03125	1.000	Excellent
26. Electrocution	5	1.00	0.03125	1.000	Excellent
27. Falls	5	1.00	0.03125	1.000	Excellent
28. Heat/Cold exposure	5	1.00	0.03125	1.000	Excellent
29. Stab/Gunshot/Penetrating trauma	5	1.00	0.03125	1.000	Excellent
30. Traffic incidents/Vehicle in water	5	1.00	0.03125	1.000	Excellent

Abbreviations: A = number of experts rating relevant; Pc = probability of chance agreement; k* = modified kappa; I-CVI = Item-Level Content Validity Index.

**Table A4:** Ambiguity.

Item	A (out of 5)	I-CVI	Pc	k*	Interpretation
1. Abdominal pain	5	1.00	0.03125	1.000	Excellent
2. Anaphylaxis/Allergic reaction	5	1.00	0.03125	1.000	Excellent
3. Animal bite	4	0.80	0.15625	0.763	Excellent
4. Back pain (non-trauma)	5	1.00	0.03125	1.000	Excellent
5. Bleeding disorder (non-trauma)	4	0.80	0.15625	0.763	Excellent
6. Breathing difficulty	5	1.00	0.03125	1.000	Excellent
7. Cardiac arrest/Death	5	1.00	0.03125	1.000	Excellent
8. Chest discomfort	4	0.80	0.15625	0.763	Excellent
9. Choking	5	1.00	0.03125	1.000	Excellent
10. Convulsion	5	1.00	0.03125	1.000	Excellent
11. Chemical hazard	4	0.80	0.15625	0.763	Excellent
12. Diabetes mellitus	5	1.00	0.03125	1.000	Excellent
13. Eye problems	5	1.00	0.03125	1.000	Excellent
14. Fever	5	1.00	0.03125	1.000	Excellent
15. Headache	5	1.00	0.03125	1.000	Excellent
16. Overdose/Poison	5	1.00	0.03125	1.000	Excellent
17. Psychiatry/Behavior change/Suicide	5	1.00	0.03125	1.000	Excellent
18. Pregnancy/Childbirth	4	0.80	0.15625	0.763	Excellent
19. Pediatric emergency	5	1.00	0.03125	1.000	Excellent
20. Sick person/Unspecify	5	1.00	0.03125	1.000	Excellent
21. Stroke	5	1.00	0.03125	1.000	Excellent
22. Unconsciousness/Fainting	4	0.80	0.15625	0.763	Excellent
23. Assault/Sexual assault	5	1.00	0.03125	1.000	Excellent
24. Burn/Blast	4	0.80	0.15625	0.763	Excellent
25. Drowning/Diving/Water-related injury	5	1.00	0.03125	1.000	Excellent
26. Electrocution	5	1.00	0.03125	1.000	Excellent
27. Falls	4	0.80	0.15625	0.763	Excellent
28. Heat/Cold exposure	5	1.00	0.03125	1.000	Excellent
29. Stab/Gunshot/Penetrating trauma	5	1.00	0.03125	1.000	Excellent
30. Traffic incidents/Vehicle in water	4	0.80	0.15625	0.763	Excellent

Abbreviations: A = number of experts rating relevant; Pc = probability of chance agreement; k* = modified kappa; I-CVI = Item-Level Content Validity Index.
